# Sebacic Acid: A Multifunctional Medium-Chain Dicarboxylic Acid in Metabolic Regulation and Tissue Regeneration

**DOI:** 10.3390/cimb48060564

**Published:** 2026-05-28

**Authors:** Luyao Qi, Jiale Feng, Xinyi Tan, Meng Yang, Lilian Ji, Weicheng Hu

**Affiliations:** 1 School of Chemistry and Life Sciences, Suzhou University of Science and Technology, Suzhou 215009, China; qiluyao__761@163.com (L.Q.); leon_xn01@163.com (J.F.); 2 School of Health and Medical Sciences, Jiangsu Food & Pharmaceutical Science College, Huai’an 223003, China; 3 School of Basic Medical Sciences, Faculty of Medicine, Yangzhou University, Yangzhou 225009, China; 19816016576@163.com (X.T.); hu_weicheng@163.com (W.H.)

**Keywords:** sebacic acid, metabolic regulation, tissue regeneration, anti-inflammatory effect, bone repair

## Abstract

Sebacic acid (SA), a ten-carbon medium-chain dicarboxylic acid, has emerged as a multifunctional bioactive metabolite with potential applications in metabolic regulation and regenerative medicine. Evidence indicates that SA exerts anti-inflammatory effects by modulating nuclear factor kappa B (NF-kB), mitogen-activated protein kinase (MAPK), and signal transducer and activator of transcription (STAT) pathways, improves glucose homeostasis by enhancing mitochondrial function and suppressing hepatic gluconeogenesis, and contributes to lipid metabolism via peroxisomal and mitochondrial β-oxidation. Beyond metabolic regulation, SA promotes bone repair by stimulating osteoblast differentiation and inhibiting osteoclast activity, and supports muscle regeneration by enhancing energy supply, cell proliferation, and the microenvironment. SA also serves as a monomer for poly (glycerol sebacate) (PGS), enabling its use in biodegradable tissue engineering scaffolds. This review synthesizes current experimental and preclinical findings on the biological functions of SA, elucidates the underlying molecular mechanisms, and highlights its translational potential for the treatment of metabolic disorders and tissue regeneration.

## 1. Introduction

Chronic inflammation [1], vascular dysfunction, dysregulation of glucose and lipid metabolism [2], and disrupted bone–muscle homeostasis [3] underlie the pathogenesis of metabolic syndrome, cardiovascular diseases, degenerative bone disorders, and muscle injuries. These conditions, often accompanied by systemic functional decline [4] and loss of internal homeostasis [5], impose significant energetic demands on key tissues—including blood vessels [6], the heart [7], skeletal muscle, pancreatic β cells, and bone-related cells. These tissues require substantial mitochondrial activity to support vascular function, metabolic balance, and insulin secretion. During functional decline or homeostatic imbalance, mitochondrial activity diminishes, leading to an energy deficit [7]. Concurrently, persistent low-grade inflammation triggered by stress, irregular diet, and lack of exercise further exacerbates this pathological state [8]. Pharmacological interventions have made significant strides in managing these interconnected conditions. For instance, metformin, a classical hypoglycemic agent, improves glucose homeostasis, while telmisartan not only protects endothelial function but also modulates glucose and lipid metabolism [9]. Despite these benefits, the efficacy of current therapies is often limited, and their long-term use may carry risks of adverse effects [10]. Consequently, natural metabolites—particularly fatty acids—have garnered attention due to their inherent safety profile and their ability to modulate multiple physiological pathways simultaneously.

Medium-chain dicarboxylic acids (MCDAs) are saturated carbon chains containing 6–12 carbons with two terminal carboxyl groups. They possess unique metabolic properties [11] and multitarget bioactivities [12]. In vivo, MCDAs are primarily metabolized in mitochondria and peroxisomes, where they contribute to energy homeostasis and glucolipid regulation. Notably, they can provide efficient energy even under pathological conditions such as insulin resistance, metabolic stress, or disorder. Recent studies have highlighted their diverse pharmacological activities, including anti-inflammatory, antibacterial, and metabolic regulatory effects. For example, azelaic acid is widely used in acne treatment, while suberic acid and its derivatives are employed in cardiovascular drug synthesis. These observations collectively underscore the potential of MCDAs in maintaining homeostasis, alleviating inflammation, and treating various diseases.

Sebacic acid (SA), a ten-carbon MCDA, exhibits excellent biocompatibility, biodegradability, and metabolic participation. Its unique chain length confers favorable molecular and material properties, making it suitable for diverse applications in physiology, disease diagnostics, biomedical materials, and chemical synthesis. Increasing evidence indicates that SA exerts multiple biological effects, including regulation of inflammation, maintenance of metabolic homeostasis, and promotion of tissue repair. For instance, SA can regulate inflammatory pathways such as MAPK and NF-κB [13]. It also serves as a metabolic substrate, participating in energy metabolism under stress conditions. Moreover, SA modulates bone metabolism balance via the crosstalk between estrogen receptors and Wnt signaling pathways [14]. Beyond its direct biological activities, SA is also widely used as a monomer for poly(glycerol sebacate) (PGS) to the development of biodegradable scaffolds for bone tissue engineering [15]. Recent evidence further shows that SA has vascular protective effects and promotes muscle regeneration. Together, these properties greatly expand its therapeutic potential.

Taken together, these features position SA as a promising candidate for the treatment of chronic metabolic and inflammatory disorders and for applications in regenerative medicine. This review systematically summarizes the sources, metabolism, biological activities, and tissue-regenerative applications of SA, with particular emphasis on its molecular mechanisms and translational potential in metabolic and inflammatory disorders.

## 2. Sources of SA

In recent years, research on SA has expanded considerably, and strategies for its preparation have become increasingly diversified. SA exists in two primary forms: endogenous and exogenous. Exogenous SA is mainly derived from natural products such as royal jelly and castor oil [16], or produced via biotechnological and chemical synthesis, whereas endogenous SA is generated through intrinsic metabolic processes in the body.

### 2.1. Exogenous SA

Regarding exogenous sources, enrichment and production strategies are relatively diverse. For example, SA represents one of the major bioactive fatty acid components of royal jelly, accounting for approximately 5% of its total fatty acid content [17]. Within royal jelly, SA occurs in two forms: free fatty acids (60–70% of total SA) and glycerol-bound fatty acid esters (30–40%) [18]. In addition, royal jelly contains 10-hydroxydecanoic acid (10-HDA), which is rapidly metabolized in the liver following ingestion to generate SA [19]. However, limitations in royal jelly yield and relatively high processing costs restrict this approach primarily to small-scale, high-value-added applications.

Compared with royal jelly extraction, SA production from castor oil via cracking is more established and industrially stable. After impurity removal and refining, ricinoleic acid, the principal component of castor oil, undergoes alkaline cleavage at high temperatures (280–320 °C), followed by sulfuric acid acidification and purification to yield SA. In traditional industrial processes, phenol was used as a diluent, and this procedure generated industrial wastewater that contributed to soil salinization and environmental pollution, thereby failing to meet modern environmental standards [20]. Recent improvements in processing technology include the use of Fe_2_O_3_ as an active catalytic material to reduce particle agglomeration and enhance cleavage efficiency. Furthermore, replacing toxic phenol with liquid paraffin as the diluent reduces reaction system viscosity, increases SA production efficiency, and improves environmental compatibility. These optimizations provide a feasible pathway for large-scale production of SA [21]. As a renewable resource, castor oil is relatively low in cost, and its processing technology is mature, with the resulting SA purity meeting industrial requirements. Therefore, this approach is well suited for large-scale industrial manufacture [22]. In addition to castor oil, trace amounts of SA are also present in coconut oil and palm oil. However, due to the complexity of processing these raw materials, extraction and purification of SA from these sources have not been widely adopted.

In addition to natural extraction and chemical synthesis, SA can also be produced through microbial catalysis. Using vegetable oils or glucose as substrates, engineered Candida tropicalis strains can oxidize these precursors to generate SA through ω-oxidation pathways. Although this method avoids phenol-related environmental pollution, a major limitation is the gradual decline in production yield after successive microbial generations. Nevertheless, this drawback can be mitigated through genetic engineering of Candida tropicalis to enhance enzymatic activity and improve SA yield [23].

### 2.2. Endogenous SA

The tricarboxylic acid cycle (TCA cycle) is closely interconnected with fatty acid metabolism and serves as a central hub for cellular energy production and biosynthesis. The TCA cycle is intricately interconnected with glycolysis, gluconeogenesis, and fatty acid metabolism, serving as a central hub linking the metabolism of carbohydrates, lipids, and proteins [24]. In contrast, ω-oxidation of fatty acids functions as a complementary or alternative pathway to β-oxidation [25]. This pathway is typically activated when β-oxidation is impaired, such as in cases of carnitine deficiency, enzymatic defects, or excessive fatty acid load.

Endogenous SA in the human body is primarily involved in fatty acid metabolism and energy production through the TCA cycle. SA is not considered a classical de novo synthesized fatty acid in humans; rather, it is mainly generated through ω-oxidation and chain-shortening reactions of precursor fatty acids during hepatic metabolism [26]. SA metabolism predominantly occurs in mitochondria, the endoplasmic reticulum (ER), and peroxisomes. Specifically, in the ER of the liver and kidneys, SA-related metabolism is catalyzed by the cytochrome P450 enzyme family. Unlike long-chain fatty acids, which require the carnitine shuttle system for mitochondrial import, SA can enter mitochondria more readily due to its partial water and lipid solubility. Its metabolism does not depend on carnitine palmitoyltransferase-1 (CPT-1) [27], the rate-limiting enzyme for mitochondrial uptake of long-chain fatty acids. Consequently, SA can bypass this transport limitation and rapidly enter mitochondria for oxidative energy production, even under conditions of altered cellular energy status [28].

The primary precursors for endogenous SA formation include long-chain fatty acids such as lauric acid, palmitic acid, and stearic acid. Within hepatic and renal microsomes, cytochrome P450 enzymes—primarily the CYP4A and CYP4F subfamilies—catalyze ω-hydroxylation of the terminal methyl group of these fatty acids, producing ω-hydroxy fatty acids [29]. These intermediates are subsequently oxidized to ω-aldehyde fatty acids. Through the coordinated action of alcohol dehydrogenases (ADH) or short-chain dehydrogenase/reductases (SDR), and aldehyde dehydrogenases (ALDH), dicarboxylic acids of the same carbon chain length are generated [30]. After release from the ER into the cytoplasm, these long-chain dicarboxylic acids undergo further β-oxidation in mitochondria and peroxisomes, resulting in progressive chain shortening and the generation of medium-chain dicarboxylic acids such as SA.

Decanoic acid (DA) is one of the MCFAs with high affinity for cytochrome P450 enzymes in liver microsomes and therefore serves as an important substrate for ω-oxidation. When defects in acyl-CoA dehydrogenase impair β-oxidation of long-chain fatty acids, ω-oxidation is compensatorily enhanced, leading to increased SA production. Enzymatic activity studies have shown that the ω-hydroxylation activity of CYP52A17 toward DA is significantly higher than that of CYP52A13, and CYP52A17 can further overoxidize hydroxydecanoic acid to SA. In contrast, CYP52A13 exhibits low oxidation efficiency toward DA and produces only small amounts of hydroxydecanoic acid, with limited capacity to generate SA. These findings suggest that the conversion of DA to SA is dependent on specific enzymatic conditions [23].

**Figure 1:**
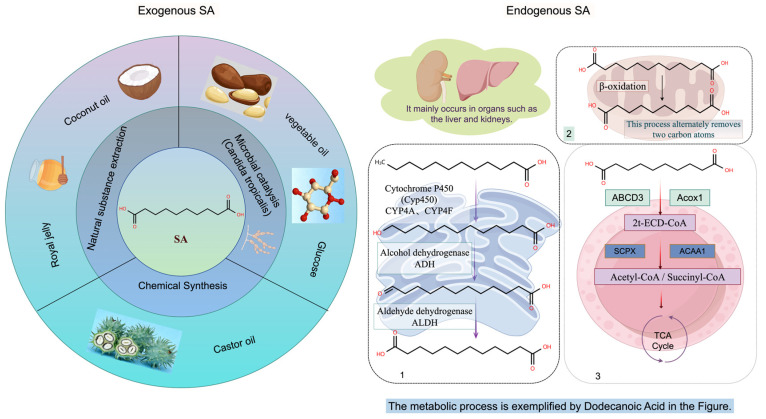
Diagram illustrating the sources of sebacic acid. The left panel shows the major exogenous sources of sebacic acid, while the right panel illustrates the primary endogenous metabolic pathway for sebacic acid production, with decanoic acid serving as a key precursor. In addition, labels 1, 2, and 3 indicate the sequential metabolic sites through which the substances pass, together with the corresponding metabolic reactions occurring at each stage.

In peroxisomes, SA enters the metabolic pathway via transport by ATP-binding cassette subfamily D member 3 (ABCD3). It is subsequently oxidized by acyl-CoA oxidase 1 (ACOX1) to form enoyl-CoA intermediates and further processed by thiolase (SCPx/ACAA1) to generate acetyl-CoA and succinyl-CoA. Succinyl-CoA can then enter the TCA cycle or participate in gluconeogenic pathways [30]. Experimental studies in rat liver mitochondria have reported that SA conversion rates reach approximately 25%, significantly exceeding those of adipic acid or octanedioic acid [26]. Overall, long-chain dicarboxylic acids undergo extension or shortening through coordinated ω-oxidation and β-oxidation in peroxisomes and mitochondria, ultimately generating SA [20]. The schematic diagram of the specific sebacic acid metabolic cycle is shown in Figure 1.

## 3. Pharmacological Effects of SA

### 3.1. Anti-Inflammatory Effects of SA

Inflammation is a fundamental defensive response of the body against tissue damage or infection, involving the coordinated action of immune cells such as macrophages and neutrophils [31], which release a cascade of pro-inflammatory cytokines (e.g., TNF-α, IL-6) and anti-inflammatory mediators (e.g., IL-10) [32]. This process is tightly regulated by several core signaling hubs, including the NF-κB [33], MAPK [34], and JAK-STAT pathways [35], as well as the NLRP3 inflammasome [36]. When the resolution phase of inflammation fails or pathogenic stimuli persist, it can lead to chronic low-grade inflammation [37]—a state characterized by persistently elevated inflammatory factors without classic clinical symptoms, which underpins the pathogenesis of numerous chronic diseases.

Initial comparative studies on the major fatty acids of royal jelly including SA, 10-hydroxy-2-decenoic acid (10H2DA), and 10-hydroxydecanoic acid (10HDAA) revealed that all three compounds concentration-dependently inhibit nitric oxide (NO) release and suppress the mRNA expression of inducible nitric oxide synthase (iNOS) and cyclooxygenase-2 (COX-2) in RAW 264.7 macrophages. However, a distinct feature of SA was its unique ability to significantly reduce the production of tumor necrosis factor-α (TNF-α), at a concentration of 5 mM. The effect not observed with the other two fatty acids [38]. Mechanistically, SA exerts this effect upstream by inhibiting the phosphorylation of p38 and c-Jun N-terminal kinase (JNK), two key kinases in the MAPK pathway that are critical for TNF-α synthesis.

SA further modulates inflammatory diseases by targeting the MAPK/NF-κB axis. At concentrations of 2.5 mM and 5 mM, it directly suppresses the transcriptional activity of NF-κB through inhibition of p65 phosphorylation. Concurrently, in a concentration-dependent manner, SA increases the protein expression of IκBα, a critical endogenous inhibitor of NF-κB. Because IκBα binds to NF-κB and prevents its nuclear translocation, SA exerts a dual regulatory effect on this pathway, thereby suppressing inflammatory progression.

At the cellular level, treatment with SA reduces interleukin-6 (IL-6) mRNA expression in LPS-activated THP-1 macrophages. At 1.5 mM, SA decreases LPS-induced IL-6 production to approximately 40% of the control level [39]. IL-6 is a central mediator in chronic inflammatory diseases, including non-alcoholic fatty liver disease (NAFLD) and rheumatoid arthritis. In these cells, IL-6 expression depends on activation of the STAT signaling axis triggered by interferon-β (IFN-β) autocrine signaling. Furthermore, SA treatment at concentrations of 0.5, 1, and 1.5 mM significantly reduces cellular IFN-β mRNA expression.

IFN-β functions as a critical autocrine cytokine that activates STAT1/3 phosphorylation via type I interferon receptors. Type I interferons are generally regulated through three principal pathways: Toll-like receptor (TLR)–mediated signaling, retinoic acid-inducible gene I (RIG-I)–like receptor–dependent signaling, and the cyclic GMP–AMP synthase (cGAS)–stimulator of interferon genes (STING) pathway [40]. These pathways ultimately converge on IRF3 and NF-κB activation, thereby initiating IFN transcription [41].

**Figure 2:**
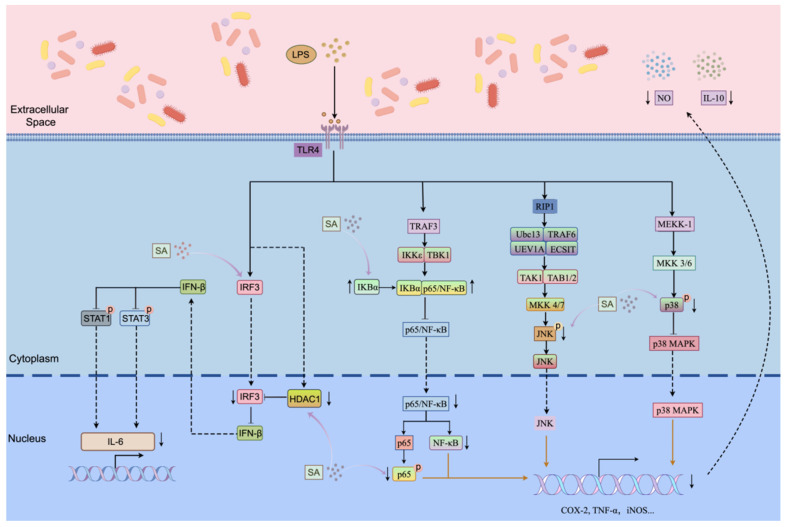
Multi-mechanistic effects of sebacic acid in the treatment of inflammation diseases.

In THP-1 cells, SA treatment has been shown to directly inhibit transcriptional initiation of IFN-β by suppressing histone deacetylase 1 (HDAC1) activity, thereby reducing nuclear accumulation of IRF3. The consequent decrease in IFN-β mRNA levels attenuates upstream STAT activation [39]. Collectively, these findings demonstrate that SA modulates inflammation by regulating IL-6 expression through the STAT pathway. Although existing studies confirm that SA directly inhibits HDAC1 activity, it remains unclear whether this inhibition occurs via increased histone H4 acetylation leading to altered chromatin accessibility at IRF3-binding sites, or through direct modulation of IRF3 acetylation. Nevertheless, these findings provide important mechanistic insights into the anti-inflammatory actions of SA. The mechanism of sebacic acid in regulating inflammation through multiple pathways is illustrated in Figure 2.

SA may also act as a metabolic biomarker for inflammatory diseases. Multiple reports indicate significant alterations in SA metabolic levels across different diseases. For instance, fecal SA levels in patients with colitis are significantly lower than those in healthy controls and show a negative correlation with disease severity [42]. In contrast, in individuals with circadian rhythm disruption (CRD), SA levels are markedly elevated and positively correlated with symptom severity. In animal models of CRD, increased abundance of *Muribaculaceae* and reduced abundance of *Akkermansia* have been observed. Treatment with CYP4A inhibitors significantly decreases SA levels in both the liver and feces, while simultaneously remodeling the intestinal microbiota, upregulating MUC2 expression in the mucus layer and tight junction proteins, and reducing endotoxin leakage. These changes collectively improve the intestinal microenvironment under inflammatory conditions [43].

Divergent trends in SA levels have also been reported in patients with diabetes, osteoporosis, colorectal cancer, and other disorders. However, the precise mechanisms underlying these metabolic alterations remain to be elucidated. As a MCDA, SA is typically generated via ω-oxidation of fatty acids, a process that may involve contributions from the gut microbiota under physiological conditions [44]. Upon entering mitochondria, unlike long-chain fatty acids, SA does not require the carnitine shuttle system. Instead, it undergoes β-oxidation to yield acetyl-CoA and succinyl-CoA, the latter of which can subsequently participate in gluconeogenesis [45]. This alternative metabolic characteristic enables SA to serve as an auxiliary energy substrate in patients with chronic metabolic disorders without exacerbating oxidative stress. In addition, SA-derived intermediates can enter the TCA cycle for ATP production. Therefore, reduced SA levels may result from gut microbiota dysbiosis, such as diminished short-chain fatty acid-producing bacteria leading to impaired fatty acid oxidation or from inflammation-induced impairment of SA absorption, thereby further exacerbating energy metabolic imbalance [46].

### 3.2. Vascular Protective Effects of Sebacic Acid

The vascular endothelium serves as more than a selective permeability barrier; it is a dynamic interface that regulates material exchange, expresses adhesion molecules to recruit inflammatory cells, and maintains coagulation homeostasis [47]. Endothelial dysfunction is widely recognized as the initiating step in the pathogenesis of atherosclerosis and a core feature of vascular aging [48], ultimately underpinning major cardiovascular diseases such as hypertension and atherosclerosis. At a mechanistic level, these pathological processes are driven by an imbalance in the vascular homeostatic regulatory network, with sustained activation of pro-inflammatory pathways—particularly NF-κB [49] and MAPK signaling—playing a central role in mediating vascular inflammatory responses and adverse remodeling.

Previous literature speculates that SA serves as one of the effector molecules responsible for its vascular protective effects. The dicarboxylate structure of SA exhibits weak acidity and lipophilicity, providing chemical active sites for subsequent copolymerization or esterification with various medicinal polymers [50]. From the perspective of vascular protection, inflammatory activation of endothelial cells is recognized as a critical step in diverse cardiovascular injuries. Inhibition of the NF-κB and MAPK signaling pathways markedly reduces endothelium-mediated inflammation and monocyte adhesion, thereby alleviating tissue damage caused by ischemia–reperfusion injury [51]. Accumulating evidence indicates that sustained endothelial MAPK activation itself drives NF-κB-dependent inflammatory stress, initiating pathology in the vascular wall and its surrounding microenvironment [52]. As previously noted, studies have demonstrated that SA inhibits both NF-κB and MAPK pathways by reducing cytokine expression, supporting the inference that its vascular protective effects are associated with these pathways.

### 3.3. Regulation of Glucose Homeostasis by Sebacic Acid

Glucose homeostasis—the dynamic balance maintained by hormonal, neural, and multiorgan coordination—is essential for supplying energy to tissues throughout the body [53]. This equilibrium is governed by key signaling networks: the PI3K/Akt pathway promotes insulin-stimulated glucose uptake by facilitating the membrane translocation of glucose transporter type 4 (GLUT4) [54], while the AMPK pathway maintains balance by activating glycolysis and suppressing gluconeogenesis [55]. Disruption of these networks leads to aberrant blood glucose levels, significantly increasing the risk of diabetes, metabolic disorders, and associated cardiovascular diseases. Consequently, the identification of safe and effective glucose-regulating agents has become a critical public health priority.

Serum metabolomics analyses indicate that SA levels are significantly decreased in patients with diabetes and insulin resistance [56]. Research demonstrates that SA is metabolized to succinyl-CoA in the liver, supplying the TCA cycle. This process dose-dependently enhances insulin-mediated glucose uptake in L6 myoblasts, accompanied by increased protein expression of glucose transporter 4 (GLUT4) [57]. Additionally, glucose uptake by peripheral tissues is enhanced. This regulatory mechanism improves insulin sensitivity, potentially alleviating hyperglycemia without necessitating weight loss [27]. These findings support the view that SA directly improves insulin resistance. Furthermore, dietary supplementation with SA in genetically diabetic db/db mice, a model of spontaneous type 2 diabetes, decreased hepatic mRNA levels of key gluconeogenic enzymes, including phosphoenolpyruvate carboxykinase (Pck1) and fructose-1,6-bisphosphatase (Fbp1), suggesting suppression of hepatic gluconeogenesis and reduced endogenous glucose output [58].

In human postprandial trials, supplementation of type 2 diabetic subjects with 10 g SA, or replacement of dietary fat with 23 g SA, reduced the postprandial incremental glucose area under the curve (AUC) by 42% and 70%, respectively. In healthy volunteers, the 23 g dose also significantly decreased glucose AUC [57]. In addition, SA lowered peripheral glucose levels. Mechanistically, SA binds to a MCDA receptor, olfactory receptor 544 (Olfr544), which exhibits highest expression in adipose tissue, liver, and intestines [59]. Ectopic expression of Olfr544 in metabolic tissues regulates cellular energy metabolism. By binding to this receptor, SA enhances mitochondrial function and promotes glucose uptake and utilization. As a MCDA, SA enters mitochondria independently of the carnitine shuttle system. Its β-oxidation produces reduced nicotinamide adenine dinucleotide (NADH) and reduced flavin adenine dinucleotide (FADH2), critical coenzymes involved in cellular respiration and energy metabolism. These coenzymes regulate glycolipid crosstalk, inhibit glycolysis, and promote fatty acid oxidation [60]. Collectively, these mechanisms represent a key pathway through which SA regulates blood glucose.

### 3.4. Modulation of Lipid Metabolism by Sebacic Acid

Lipid homeostasis—the precise regulation of cholesterol and triglyceride synthesis, transport, uptake, and clearance—is essential for vascular health. Disruption of these regulatory mechanisms leads to lipid deposition in the vessel wall, oxidative modification, and sustained inflammation, ultimately driving the pathogenesis of atherosclerosis and its complications [61]. Given the well-established metabolic crosstalk between glucose, fatty acids, and cholesterol, metabolites that participate in core energy cycles—such as the TCA cycle—are poised to exert significant influence on systemic lipid profiles [62].

Studies in obese mouse models demonstrated that after six weeks of aloe-emodin feeding, urinary levels of various organic acids, including SA, returned to normal [63], suggesting a relationship between SA and blood lipid reduction. Within cells, SA serves as a non-storable alternative fat source, preferentially metabolized via peroxisomal and mitochondrial pathways to generate acetyl-CoA, succinate, and other intermediates, which replenish the TCA cycle for rapid energy production [11]. This pathway enhances fatty acid oxidation and reduces hepatic synthesis of triglycerides and cholesterol [64]. Beyond energy provision, SA as one of the main components of royal jelly reduces liver fat accumulation, particularly saturated fatty acids [65], offering potential benefits for energy metabolism and obesity mitigation [11].

At the gene regulation level, at a concentration of 0.5 mM, SA downregulates the expression of fatty acid metabolism-related and fibrosis-related genes in hepatocytes, thereby reducing lipid droplet formation [66]. In HepG2 cells, Oil Red O staining revealed a significant reduction in lipid-positive area in palmitic acid-induced lipid-accumulating cells following SA treatment. SA also decreased mRNA expression of lipogenic genes (FASN and SCD1) and the fibrosis-related gene COL1A1, with a reduction of approximately 50% compared with the model group, indicating suppression of both lipogenesis and hepatic fibrosis progression. In the same model, SA at 0.5–1.5 mM downregulated hepatocyte nuclear factor 4α (HNF4α), inhibiting the expression of lipid regulator ANGPTL8 [67], leading to reduced hepatic cholesterol synthesis. Increased SA intake enhanced fecal excretion of saturated fatty acids and reduced intestinal lipid absorption, indirectly lowering total cholesterol (TC) and LDL-C [66]. It provides direct evidence of lipid-modulating effects of SA.

The ALT/AST ratio is a crucial indicator of liver function. Animal and human studies demonstrate a negative correlation between plasma SA concentration and liver aging, with lower levels in aged rats and elderly humans compared to younger counterparts. SA supplementation delays hepatic telomere shortening and improves liver enzyme profiles [68]. In high-fat diet-induced NAFLD rats, feeding royal jelly containing SA reduced hepatic TG and restored AST and ALT activities, indicating improved liver function. Notably, SA-treated animals exhibited only a mild increase in AST within the normal range, showing no significant toxicity.

Despite these promising findings, several critical questions remain. Most studies to date have utilized SA as a metabolic biomarker rather than a therapeutic intervention, and human data on its lipid-lowering efficacy via direct supplementation are limited [69]. The hypothesis that age-related accumulation of SA contributes to its metabolic effects requires direct experimental validation. Future research should prioritize well-designed clinical trials to establish dose–response relationships, evaluate long-term safety, and explore potential synergies with existing lipid-lowering therapies. Nonetheless, the multifaceted mechanisms and favorable safety profile of SA position it as a compelling candidate for further development in the management of dyslipidemia and its cardiovascular sequelae.

### 3.5. Role of Sebacic Acid in Bone Repair and Regeneration

Bone is a dynamic tissue whose structural integrity and homeostatic balance depend on the precise coupling of bone formation and resorption [70]. This equilibrium is primarily orchestrated by two cell types: osteoblasts, which secrete organic matrix molecules to form new bone, and osteoclasts, which resorb bone matrix. Disruption of this delicate balance—whether by aging, inflammation, or metabolic dysfunction—leads to microarchitectural deterioration and the development of common bone disorders such as osteoporosis [71].

SA demonstrates a dual mechanism in treating bone-related disorders. First, it acts directly as a bioactive molecule to promote osteoblast differentiation, enhancing expression of functional markers such as ALP and suppressing osteoclast activity, thereby modulating bone metabolic balance. Second, SA serves as a monomer for biodegradable polymers such as PGS [72]. These polymers enable construction of bone tissue engineering scaffolds with excellent biocompatibility and controllable degradability, providing a favorable microenvironment for osteocyte growth and new bone formation, thereby promoting repair and regeneration.

SA promotes osteoblast function directly. In KS483 osteoblasts, SA induced formation of mineralized nodules, an effect inhibited by the estrogen receptor (ER) antagonist ICI182780, indicating an ER-dependent mechanism. Molecular docking suggests SA can bind to the coactivator site or dimerization region of ERα, potentially influencing dimerization or coactivator recruitment. This mechanism may provide a new strategy for treating bone metabolic diseases without stimulating cancer cell proliferation [14].

A recent study showed that SA enhances ALP activity and mineralization in MC3T3-E1 osteoblasts, reflecting osteoblast maturity and function [73]. Similar effects were observed in primary osteoblast-related cells (ST2, POBs, BMSCs), suggesting universal osteogenic activity [74]. SA also upregulated expression of osteogenic marker genes across differentiation stages, including early markers Runx2 and Osterix (Osx), mid-stage markers Col1A1, ALP, and bone sialoprotein (Bsp), and late-stage osteocalcin (Ocn). Calcein and Alizarin Red staining confirmed enhanced mineralized nodule formation. Since the Wnt/β-Catenin pathway regulates Runx2/Osx transcription and ALP activity [75], SA may exert effects via this signaling axis.

SA also modulates osteoclast activity. Osteoclast differentiation, primarily regulated by RANKL and M-CSF via NF-κB and MAPK pathways [76], contributes to inflammatory bone diseases such as rheumatoid arthritis and osteoporosis. SA inhibits TNF-α and LPS-induced IL-6 expression in macrophages, suggesting potential suppression of osteoclast differentiation via modulation of MAPK/NF-κB pathways. While mechanistic details remain under investigation, SA’s osteogenic potential has been initially confirmed, providing a candidate for natural bone repair therapeutics.

PGS, synthesized from glycerol and SA [77], exhibits bioelasticity and biocompatibility, avoiding severe immune rejection. Its mechanical properties provide temporary support for new tissue [78], and controlled degradation obviates secondary surgery. As a degradation product, SA itself exerts osteogenic effects. PEG-modified PGS hydrogel (PEGS) restores osteogenic capacity in aged bone marrow mesenchymal stem cells by scavenging ROS [79].

### 3.6. Role of Sebacic Acid in Muscle Regeneration and Injury Repair

Skeletal muscle regeneration is a complex physiological process initiated in response to myofiber injury. It depends not only on the proliferation and differentiation of muscle satellite cells but also on the coordinated participation of other cell types, including immune cells and fibroblasts, which collectively orchestrate the repair microenvironment [80]. This process is energetically demanding and tightly regulated by cytokines and multiple signaling pathways [81]. Disruption of this microenvironment—whether by aging, chronic inflammation, or metabolic stress—is a major contributor to the decline in muscle regenerative capacity [82].

SA influences muscle regeneration through energy supply, cell proliferation, and microenvironment modulation. As a MCDA, it enters mitochondrial β-oxidation directly, supplying rapid energy during high-intensity exercise or stress (sepsis, burns), thereby reducing muscle protein breakdown and lactate accumulation [45]. Animal studies confirm improved endurance and lower serum urea nitrogen and lactate following oral SA administration [71].

Serum SA levels decrease with age and correlate positively with skeletal muscle ATP levels. In senescent C2C12 myoblasts, SA (62.5 μM) enhances proliferation [83] and upregulates Cyclin D1 and Cyclin A2. SA also mimics IGF-1/IGFR pathway activation, promoting myogenesis. In C2C12 cells, six-day SA treatment increased the fusion index of MyHC-positive myotubes from 15% to 45%, elevated myogenin levels, and enhanced myotube length and diameter, demonstrating promotion of both proliferation and differentiation [84].

**Figure 3:**
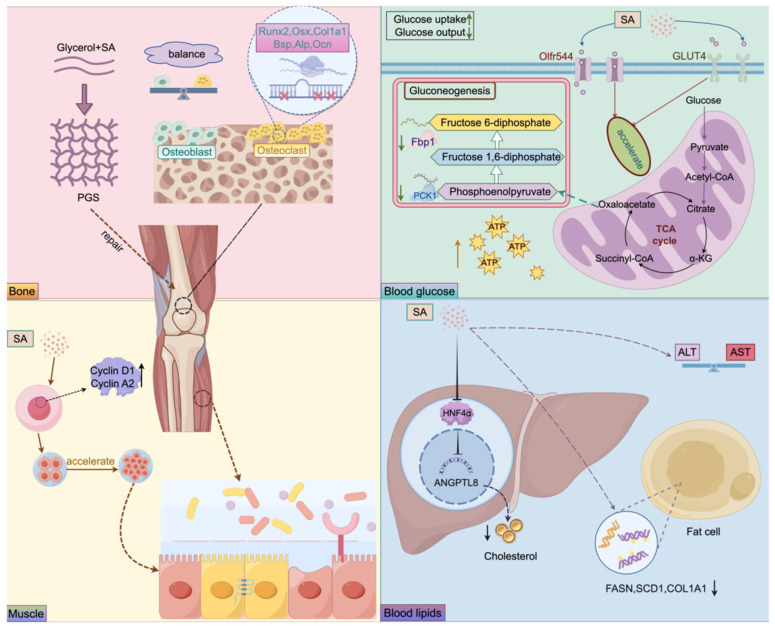
Regulatory roles of sebacic acid in disease-relevant mechanisms. MC3T3-E1 osteoblasts were used as a model for bone metabolism; L6 myocytes were used for glucose regulation; senescent C2C12 myoblasts were used for muscle-related studies; and HepG2 hepatocytes were used as a model for lipid metabolism. This figure summarizes the SA-associated regulatory mechanisms reported in the aforementioned studies.

The detailed mechanisms by which sebacic acid exerts its effects on bones, muscles, blood glucose, and blood lipids are illustrated in Figure 3. Table 1 summarizes the regulatory pathways and related factors involved in all disease mechanisms associated with azelaic acid as mentioned above.

## 4. Conclusions

This review systematically and comprehensively summarizes the current research advances of SA in three core biological functions, including its regulatory role in inflammatory signaling pathways, its maintenance of glucose and lipid metabolic homeostasis and its involvement in skeletal tissue repair and regeneration. Beyond these known biological activities, more research evidence is redefining the biological role of SA, identifying it as a critical endogenous signaling molecule and a key modulator of host-microbe interactions, which is essential for fully elucidating its therapeutic potential. Furthermore, the translational application of SA in the development of biodegradable scaffolds for bone tissue engineering, as well as its emerging roles in vascular protection and muscle regeneration, highlights its dual value as both a bioactive metabolite and a monomer for functional biomaterial synthesis.

## 5. Future Perspectives

Future research on SA should focus on its endogenous biosynthesis rather than exogenous intervention. Key priorities include elucidating SA metabolic mechanisms under different pathological conditions, identifying its molecular targets, and clarifying its interactions with the gut microbiota. Particular attention should be given to biosynthetic pathways involving the coordinated metabolism of dietary precursors by host cytochrome P450 enzymes and gut microbes. Multi-omics and synthetic biology approaches may help develop dietary or probiotic strategies to precisely regulate endogenous SA production. Such strategies may support precision management of metabolic health and further enhance the therapeutic potential of SA. In addition, further studies are needed to validate the in vivo efficacy of SA in preclinical models. Future work should also clarify its mechanisms of action and molecular targets.

## Figures and Tables

**Table 1:** The regulatory roles of sebacic acid in various disease models and related mechanisms.

No.	MCDA	Study Type	Experimental Model	Dose	Mechanism of Action	Refs.
1	SA	in vitro	LPS-stimulated RAW 264.7	1, 2.5, 5 mM	↓ TNF-α, IL-6, IL-10, iNOS, COX-2, HO-1, p38, p65, JNK1/2, NF-κB ↑ IκBα	[38]
2	SA	in vitro	LPS-stimulated dTHP-1	0.5, 1, 1.5 mM	↓ IL-6, p-STAT1/STAT3, IFN-β, HDAC, IRF3	[39]
3	SA	in vitro	L6 myoblasts	0.2 mM	↑ GLUT4 ↑ glucose uptake	[57]
4	SA	in vitro	PA-induced HepG2	0.5 mM	↓ FASN, SCD1, COL1A1	[66]
5	SA	in vitro	HepG2	0.5, 1, 1.5 mM	↓ ANGPTL8, HNF4α	[67]
6	SA	in vitro	MCF-7, HeLa, Huh7 and KS483 cells	0.0001–10 μM	↓ E2-induced ERα/ERβ recruitment, ↓ pS2 mRNA, ↓ ERα-EAB1 interaction ↑ ERβ recruitment, ↑ ERα transcriptional activity, ↑ osteoblast mineralization,	[14]
7	SA	in vitro	Glycerophosphate and ascorbic acid-induced MC3T3-E1	10 μM	↑ Runx2, Osx, Col1a1, Alp, Bsp, Ocn ↑ Mineralization ability ↑ ALT activity	[74]
8	SA	in vitro	D-galactose-induced C2C12	15.6, 31.2, 62.5, 125 μM	↑ Cell viability	[83]
9	SA	in vitro	C2C12	500 μM	↑ Cyclin D1, A2, Myogenin, MyHC proteins	[84]
10	SA	in vivo	SSW mice	77.6 g/kg	↓ Defb37, Defb40, Camp ↑ Muc2, Fcgbp, Clca1, Zg16, Retnlb	[43]
11	SA	in vivo	db/db mice	7.76 g/kg, 77.6 g/kg	↓ Pck1, Fbp1 ↑ SCD1, PPARγ, LPL	[58]

SA: Sebacic acid; ↑: Upregulate; ↓: Downregulate.

## Data Availability

No new data were created or analyzed in this study. Data sharing is not applicable to this article.
